# Mouse Models of Prostate Cancer

**DOI:** 10.1155/2011/895238

**Published:** 2011-02-23

**Authors:** Kenneth C. Valkenburg, Bart O. Williams

**Affiliations:** Van Andel Research Institute, 333 Bostwick Avenue N.E., Grand Rapids, MI 49503, USA

## Abstract

The development and optimization of high-throughput screening methods has identified a multitude of genetic changes associated with human disease. The use of immunodeficient and genetically engineered mouse models that mimic the human disease has been crucial in validating the importance of these genetic pathways in prostate cancer. These models provide a platform for finding novel therapies to treat human patients afflicted with prostate cancer as well as those who have debilitating bone metastases. In this paper, we focus on the historical development and phenotypic descriptions of mouse models used to study prostate cancer. We also comment on how closely each model recapitulates human prostate cancer.

## 1. Introduction

The American Cancer Society estimates that approximately 218,000 men will be diagnosed with, and 32,000 men will die of, prostate cancer in the United States in 2010 [1]. To give these numbers context, prostate cancer (PCa) is the most commonly diagnosed cancer in men and is responsible for the second highest number of cancer-related deaths in men in the United States. PCa represents 27.6% of new cancer cases in men and 10.7% of cancer-related deaths in men [1]. An estimated 1 in 3 men will be diagnosed with PCa or a precancerous prostatic lesion in their lifetime [2]. In Europe, there are approximately 346,000 new PCa cases and 87,000 deaths per year [3]. 

Age is the main risk factor for prostate cancer; between 2002 and 2006, the median age at diagnosis was 68, and the median age at death was 80 [4]. Approximately 95% of men over the age of 70 present with prostatic hyperplasia [5]. Though many men are afflicted with PCa, most will not die of the disease. Surgery, radiation therapy, and androgen deprivation therapy have increased survival rates. “Watchful waiting” is also an option for patients (especially those over the age of 75) who have more indolent disease and who might not benefit from intense treatment [4]. Upon diagnosis (via biopsy following a digital rectal exam (DRE) or a prostate-specific antigen (PSA) screen) clinicians generally base treatment on Gleason score [6] and tumor stage. The five-year survival rate PCa patients after diagnosis at local stages is 100%, but for metastatic disease, it is only 30.6% [7].

PCa is largely dependent on androgens for growth and proliferation; hence, androgen deprivation therapy (chemical castration) is the standard of treatment, and it generally causes prostate tumors to regress. However, most PCa cases eventually recur after treatment. These more lethal cases generally have a high Gleason score and can be metastatic and/or refractory to androgen deprivation therapy (castration resistant). Skeletal metastasis is the most significant cause of morbidity and mortality in PCa [8]. Skeletal metastases are found in approximately 90% of patients who die of PCa [9]. This adds to the health burden of these patients while they are still alive, due to painful lesions that impair mobility and cause pathologic fractures, spinal cord compression, and symptomatic hypercalcemia [10]. The frequency of bone metastases in PCa indicates that the microenvironment of the bone may promote the growth of PCa cells. The proportion of active osteoblasts is usually greater than that of active osteoclasts in PCa bone metastases, resulting in the net formation (rather than lysis) of bone in a majority of these lesions [11]. However, osteolysis is required for metastatic tumor cells to invade the bone matrix. Also, patients with bone metastases have a higher risk of fracture, indicating that bone destruction is occurring. One study suggests that there may be more bone lysis during prostate tumor metastasis than originally thought and that it might be more prevalent in PCa than other diseases [12]. While much work has been done in the area of prostate tumor-bone crosstalk [13–15], there is still much to learn in this area. Accordingly, there is an urgent need for new treatments and better experimental models for the study of PCa development, progression, and metastasis. 

PCa is a heterogeneous disease in which malignant cells arise from the epithelial layers of the prostate. These layers comprise luminal secretory cells, basal cells, and rare neuroendocrine cells. A diagnostic feature of PCa is a luminal phenotype and the loss of differentiated basal cells [16]. A debate is ongoing as to which type of epithelial cell represents the cell of origin for PCa: luminal stem cells [16], basal stem cells [17], or both [18]. Given the heterogeneity of the disease and the many genetic pathways that are involved, there is likely a complex explanation [19]. An understanding of where malignant cells arise from in the prostate may be of vital importance for the development of more effective treatments [20]. Mouse models may provide valuable insight into this challenging question. 

Mouse modeling has made a significant contribution to the study of prostate development and disease. This paper reviews the history and development of some of the most common mouse models of PCa and discusses the genetic alterations that were used to make the models, as well as the clinical implications of each model. It is important to note that the genetic backgrounds of the models are outside the scope of this paper; this topic has been reviewed elsewhere [21].

## 2. Nonmouse Models

Immortalized cell lines have been used extensively to study various aspects of PCa, particularly in terms of genetic deregulation. In 2005, a two-part review was written examining PCa cell lines in detail [22, 23]. Another article characterized 17 cell lines so as to simplify the selection process for research [24]. An issue with cell lines is that they are commonly derived from metastatic lesions, therefore precluding analysis of genetic alterations that transform normal prostate cells to malignant cells. However, while care must be taken in extrapolating from *in vitro* data to *in vivo* meaning, PCa cell lines provide a model for identifying prospective gene targets in a fast and efficient manner, as well as for determining molecular mechanisms. Primary cell cultures, taken directly from tumor tissue, also provide a model for studying various aspects of PCa [25]. The development of improved *in vivo* models should allow researchers to extend the insights gained from the decades of cell culture work. 

PCa occurs naturally in dogs and in some strains of rats [26]. The dog most closely resembles humans in terms of PCa characteristics [27]. PCa in the dog metastasizes to bone in an osteoblastic manner in 24% of cases [28]. Canine PCa is also age dependent. While dogs may seem an ideal model for studying PCa, there are limitations to their use. The instances of PCa do not diminish in castrated dogs, indicating that tumor growth is not androgen dependent [28]. There is also a relatively long latency in dogs. The high cost, long gestation period, and difficulty of genetic manipulation make the dog an unrealistic experimental model. 

Several strains of rats, including the Dunning, Copenhagen, and Wistar rats, have been well characterized, and they develop a wide range of cancer phenotypes in the prostate [29–31]. However, due to the rarity of tumors, variability in phenotypes, long latency periods, and lack of metastases, the realistic probability of using them as models is low. Three articles have recently been published describing different methods of generating knockout rats [32–34]. This indicates that the use of the rat as a genetically engineered model could increase in the coming years [35].

## 3. Prostate Cancer Mouse Models

### 3.1. Pros and Cons of Mice in Translational Research

**Figure 1:**
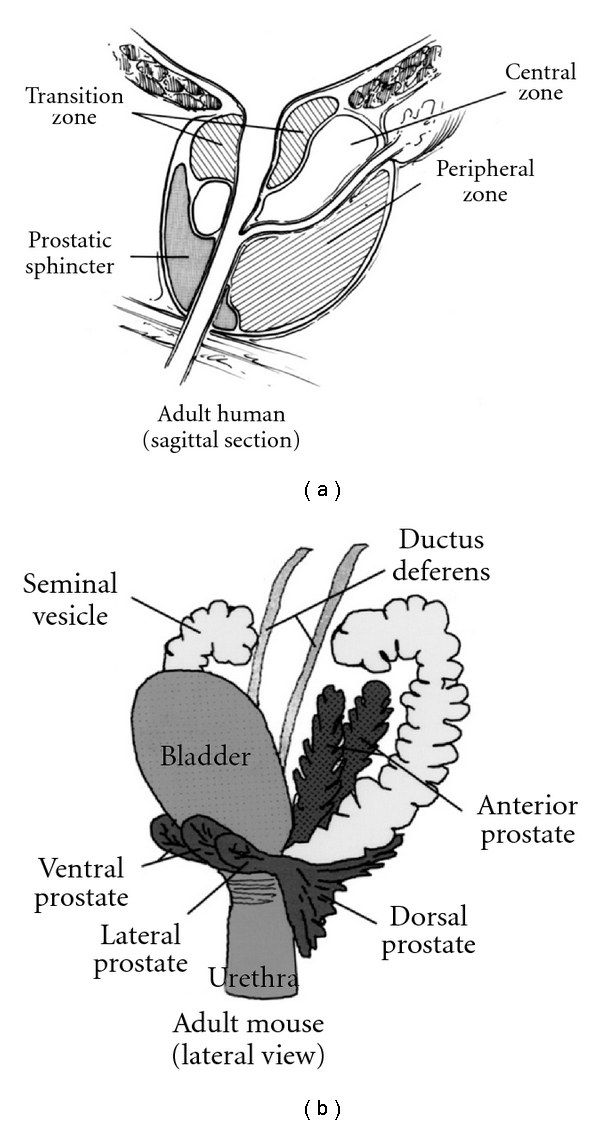
Schematic illustration of the anatomy of the human prostate (a) and mouse prostate (b) (adapted from McNeal [42] and Cunha et al. [43], resp.). Used with permission: Abate-Shen and Shen [2].

Naturally occurring PCa is uncommon in the mouse; after a two-year study by the National Toxicology Program of 612 mice, there were no spontaneous cases of carcinoma in the prostate [36]. Therefore, a great deal of work has been done to manipulate mice so that they develop PCa that accurately recapitulates human disease. Mouse and human prostate anatomy is dissimilar. The mouse prostate has a lobular structure with four lobes—anterior (also known as the coagulating gland), ventral, dorsal, and lateral (these last two lobes are commonly referred to as the dorsolateral lobe) [37]. Alternatively, the human prostate has one “lobe” divided into three zones: central, transitional, and peripheral (Figure 1). The majority of human PCa is found in the peripheral zone, which comprises about 75% of the tissue in the prostate. The mouse dorsolateral lobe has been described as the most similar to the human peripheral zone [38], but the consensus opinion of the Bar Harbor Pathology Panel is that there is no direct relationship between any one mouse lobe and any of the zones of the human prostate [36]. An additional concern is that the histopathology and time-frame of prostatic disease development can be different in mice [36]. Also, the lifespan of a mouse is 30–50 times shorter than that of humans, and mice are about 3,000 times smaller [39]. This means, for example, that pharmacokinetics during drug studies tends to differ between mice and humans due to the size difference [40]. Cancer metastases in mice have a propensity to originate from mesenchymal cells whereas human metastases generally originate from epithelial cells, especially in PCa [41]. Finally, it has proven difficult to induce bone metastases in mice, which is a problem because this type of metastasis is common in human PCa patients [13]. All of these factors provide challenges for mouse researchers and must be considered when extrapolating research conclusions from mice to humans. 

Despite the several concerns, the mouse is still one of the best animals in which to model human cancer. First, mice are as susceptible to cancer as humans [39]. However, the type of cancer that mice are afflicted with is not always reflective of that in humans; mice tend to have more sarcomas and lymphomas and fewer carcinomas [44–46]. This may be due to differences in relative telomere length and function [46]. Second, the mouse and human genomes are approximately 95% identical, and mice have many structurally similar genes and genomic alterations that have been implicated in cancer [40, 47]. Third, mice are relatively easy to genetically modify, especially with the Cre-loxP system (reviewed later in this paper). Finally, because mice have a relatively short gestation time and are small, they are reasonably easy and affordable to house and breed to generate large populations. 

**Figure 2:**
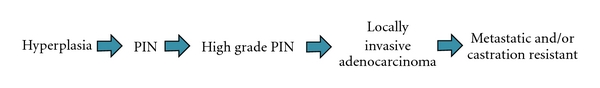
Stages of prostate cancer. The goal of genetically engineered mouse models is to accurately mimic all of these stages of human disease in the mouse.

The goal of every mouse model is to accurately imitate human disease so that molecular mechanisms can be found and new therapies can be tested. An example of the successful use of a mouse model in translational cancer research is the modeling of the BCR-ABL translocation and the efficiency of the kinase inhibitor Gleevec to treat acute myelogenous leukemia (AML) [48]. The ideal PCa mouse model would exhibit hyperproliferation and hyperplasia in prostate epithelial cells leading to prostatic intraepithelial neoplasia (PIN), which is a noninvasive precursor to PCa (Figure 2). The affected prostate would then develop noninvasive carcinoma in situ, commonly known as high-grade PIN (HGPIN). The next stage would be locally invasive adenocarcinoma, followed by metastatic disease and/or castration resistance. Because human PCa most often metastasizes to the skeleton and lymph nodes, these would be the most ideal sites in the mouse. During the stages listed thus far, the ideal model would display androgen-dependent disease. It would be beneficial for the ideal model to eventually develop castration-resistant disease, comparable to the human disease that is currently untreatable. The histopathologic features and molecular pathways that are changed in humans should also be changed in the mouse. Finally, the response of the ideal model to therapeutics would accurately reflect the response in humans. More ideas about limitations and goals of modern mouse models can be found in a review from 2008 [49].

### 3.2. Five Main Categories of Prostate Cancer Mouse Models

Over the past few decades, numerous PCa mouse models have been developed, studied, and characterized. We will discuss some of the most relevant models in the field within five categories. The first category comprises the xenograft models, of which there are many kinds. The final four categories fall under the broad class of genetically engineered mouse models (GEMMs). These include transgenic models that use prostate-specific promoters to express the SV40 T antigen, as well as those expressing other oncogenes. The fourth and fifth categories are traditional and conditional knockout models. 

#### 3.2.1. Xenograft Models

A common *in vivo* PCa model is the immunodeficient mouse as a recipient of human tumor tissue, cell lines, or primary cell cultures. These mice are used because of their inability to mount an immunologic response to foreign (i.e., human) tissue, allowing human tumors to grow relatively unabated. These systems are responsible for important insights into the mechanisms underlying many human tumors, and they allow for propagation and expansion of patient-specific material. Human samples can be serially transplanted in parallel to numerous individual mice so that the efficacy of specific treatments can be evaluated. This approach holds promise for identifying the most effective treatments for human patients [50]. These models have also been used to determine stem cell-like properties of cancer cells. The number of models using a xenograft system is potentially endless because of the multitude of genetic alterations and combinations (as well as the wide range of human tumor tissue) that can be grafted into immunodeficient mice. Therefore, we will discuss the most common immunodeficient mice and their relative lack of immunity and then discuss several examples of PCa-specific models that were generated using these mice with the caveat that there are numerous other xenograft models described elsewhere.

**Nude Mice**

The first xenotransplantation of human PCa tissue, the androgen-responsive PC-82 tumor model, was demonstrated in 1980 in athymic nude mice on a BALB/c genetic background [51]. Nude mice are deficient in T lymphocytes due to lack of a thymus, so they cannot mount an immunologic response to foreign tissue. The rate of successful xenografts was approximately 3% until 1996, when seven new xenograft models were described at a take rate of 38% using the newer NMRI nude strain (developed at the Naval Marine Research Institute) [52]. Since that time, many different models have been developed using athymic nude mice [53]. An example is a model that demonstrated the ability of a xenograft to metastasize to the lymph node and axial skeleton [54]. In this model, cells from the C4-2 PCa cell line (a castration-resistant subline of the LNCaP cell line) were injected subcutaneously at different sites, as well as orthotopically into the dorsolateral prostate lobe. Bear in mind that orthotopic implantation has been considered a more accurate representation of the original disease, due to a more accurate representation of the microenvironment of the growing tumor [55]. This model resembled human PCa in that the metastases were found in the lymph node and bone, the two most common sites of human PCa metastasis [54].

**SCID Mice**

Severe combined immunodeficiency (SCID) is a rare disease in which the affected organism is unable to mount an immune response due to loss of B and T lymphocytes. Mice with an autosomal recessive SCID mutation were characterized in 1983 [56]. SCID mice are deficient in mature B and T cells, due to a defect in genetic recombination necessary for lymphoid development [57]. However, natural killer (NK) cells and myeloid cells appear normal in SCID mice [57, 58], and some SCID mice are “leaky,” meaning that some B and T cells are still present. Therefore, some SCID mice will reject foreign tissue [59]. SCID mice were used in a model in which the tyrosine kinase HER2/neu was overexpressed in LNCaP cells (an immortalized PCa cell line), which were subcutaneously injected into the mice. The goal of this model was to determine if HER2/neu could induce androgen-independent growth of tumors. This was shown to be true and was due to modulation of the androgen receptor (AR) signaling pathway [60]. Another model is the SCID-hu (SCID-human) in which human fetal bone is implanted subcutaneously into the flank of the mouse [61]. In this model, PCa cells (which can be genetically modified to suit the experimental hypothesis) are injected into the tail vein of the mouse, and the ability of these cells to metastasize to the human bone is measured and compared [61].

**NOD-SCID Mice**

**Figure 3:**
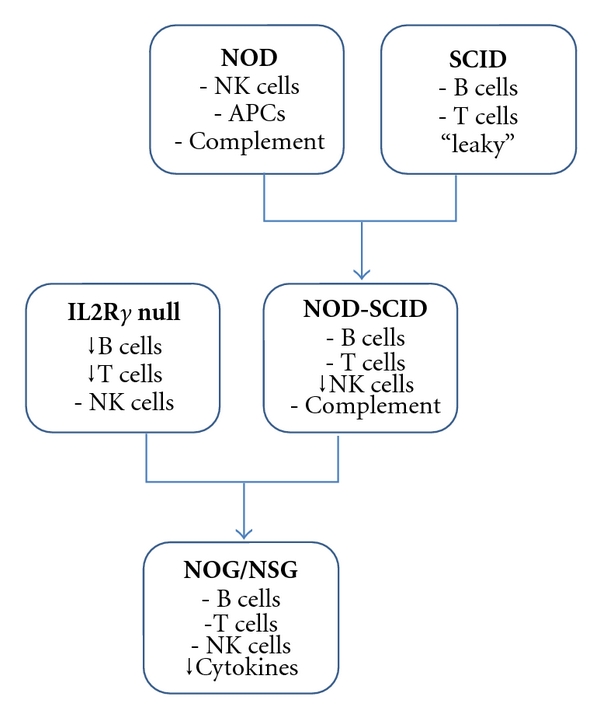
Schematic diagram of the cross-breeding of immunodeficient mice. Note: “-” or “↓“ could refer to absence or reduction in number of cells or in activity.

To improve the immunodeficient properties of the SCID model, SCID mice were crossed to nonobese diabetic (NOD) mice, which are deficient in NK cells, circulating complement, and functional antigen-presenting cells (APCs) [62, 63]. NOD-SCID mice were able to accept foreign tissue at a higher success rate and were more immunodeficient than SCID mice (Figure 3) [64]. However, there is evidence of remnant NK cell activity in these mice [65]. Two years after the PCa SCID-hu model was described [61], similar work was done in the NOD-SCID model [66]. Human adult bone was implanted into the mice subcutaneously, and PCa cells were injected into the tail vein. Metastasis to the bone occurred at a higher rate in the NOD-SCID mice (13/20 mice) relative to SCID mice (5/19 mice) [61, 66].

**NOG/NSG Mice**

Another improvement of the immunodeficient mouse occurred when NOD-SCID mice were crossed to interleukin 2 receptor *γ* (IL2R*γ*) null mice (also known as X-SCID mice) which are completely lacking NK cells [67–69]. This model is generally referred to as the NOG or NSG model, and it is currently the most severely immunodeficient mouse available [70, 71]. These mice have a complete absence of B, T, and NK cells, have a deficiency in cytokine signaling, and show no phenotypic “leakiness” after a year of age. NOG/NSG mice have a higher xenograft success rate than NOD-SCID mice (13/13 compared to 8/13, when human hematopoietic stem cells were injected) [71]. NOG mice also survived longer than NOD-SCID mice (median of 89 weeks compared to 37 weeks [71]) making them a more valuable model, especially for long-term studies. The use of the NOG/NSG mouse over other immunodeficient mice can make a drastic difference in experimental results, as shown in an article that reported differing results after performing limited dilution analysis in NOD-SCID and NOG/NSG mice [72]. A recent article describes the effectiveness of the NOG/NSG mice over nude mice [73]. In this study, PCa cells were injected subcutaneously into nude and NOG/NSG mice. Palpable tumors grew in all of the mice, but after 6–16 weeks, the tumors stopped growing and regressed in the nude mice whereas the tumors in the NOG/NSG mice grew at an accelerated rate and did not regress [73]. This illustrates the utility of growing xenografts in NOG/NSG mice.

**RAG Mice**

RAG mice are deficient in the recombination activating gene (RAG). Two proteins, RAG1 and RAG2, are synergistically responsible for activation of V(D)J recombination during T cell development [74, 75]. When either of these proteins is inactivated, the mice are deficient in both B and T cells, similar to SCID mice [76, 77]. These mice also have an inflammatory response and NK cell activity. A PCa-specific model using these mice was reported in 2006, when castration resistant TRAMP-C2 cells were subcutaneously injected into RAG1 null mice to ascertain effectiveness of a specific antitumor treatment [78].

**Renal Grafts and Intrabone Injections**

Renal grafting and intrabone injections are two *in vivo* model systems that merit further discussion. Renal grafting is the process of recombining PCa cells with rat urogenital sinus mesenchyme (UGM) cells and then transplanting this recombinant tissue beneath the kidney capsule in an immunodeficient mouse (historically nude or SCID) to assess growth and other phenotypes [79]. The recombining of prostate cells and UGM cells was first described in 1978, the purpose being to determine the importance of the mesenchyme for androgen dependency [80]. This procedure is currently most commonly used to determine the ability of putative prostate stem cells to generate prostatic tissue and ducts. This model has been used to show that castration resistant Nkx3.1-expressing cells (CARNs) are putative prostate luminal epithelial stem cells [16]. Another study used this system to show that a single Lin^−^Sca1^+^CD133^+^CD44^+^CD117^+^ cell could regenerate substantial prostate ductal structures [81].

Another purpose for renal grafting is to determine the physiological significance of genes that cannot be studied via whole body knockouts due to embryonic lethality (this is called “tissue rescue”). One example of this is the Rb-null mouse. Rb^−/−^ mice die after 13 days of gestation, so the physiological role of Rb in the prostate could not be adequately determined in adult male mice. Consequently, pelvic organ rudiments from Rb null mice were grafted under kidneys of adult male nude mice [82]. The grafts formed well-differentiated epithelial prostate ductal structures. Mice containing wild-type (WT) Rb grafts and mice containing null Rb grafts were both treated with testosterone propionate and estradiol to induce carcinogenesis. Upon treatment, both WT and null grafts displayed hyperplasia, but only the Rb null grafts developed atypical hyperplasia and cancer. This model validates the tumor suppressive activity of Rb in the prostate, which would not have been possible using the whole body knockout model. Another example of tissue rescue from an embryonically lethal mouse is the p57(Kip2) model [83]. Urogenital tissue was microdissected from p57 knockout mice and grafted under the kidney of nude mice [84]. The tumors that resulted had many similarities to human PCa, especially in terms of the kinetics of tumor development and differentiation patterns. This model was not only important to determine p57's role in PCa but also may be an easily adaptable model for future molecular studies.

Intratibial and intrafemoral injections have been used to model the invasion and growth of PCa cells in bone, providing a platform for studying bone microenvironment and bone-tumor crosstalk, which is essential to understanding why PCa tumors so frequently metastasize to the skeleton. The reason these models are so valuable is because there is no mouse model that spontaneously metastasizes to bone. Intratibial injections were first described for PCa in 2002, when cells from three PCa cell lines were injected into the tibiae of nude mice to compare their relative ability to invade and grow in bone [85]. Cells may also be genetically altered to study the effect of specific genes on the ability of cells to grow in bone. This was demonstrated in 2005 when the Wnt antagonist DKK1 (Dickkopf 1) was knocked down using short hairpin RNA (shRNA) in PCa cell lines, and these cells were then injected into the tibiae of SCID mice [86]. Intrafemoral injections can also be used in the same context as intratibial injections. Femurs are larger than tibiae in overall size and cavity size, so it depends on the situation as to which bone is chosen for injection. As an example, Fizazi et al. injected human MDA PCa 2b cells into femurs of SCID mice to determine the mechanism by which these cells form osteoblastic lesions in bone [87]. Intrabone injections represent an important model for the elucidation of the importance of genetic pathways and other factors in PCa metastasis to bone.

**Summary 3.2.1**

Despite the utility of the xenograft systems, there are limitations to their use [49]. A compromised immune system, while it is required to allow growth of foreign tissues in mice, raises concerns about how accurately these mice model tumor progression. The interaction between immune cells and tumor cells may play an important role in human PCa metastasis [88], and the metastasis that takes place in the absence of an immune system may be misleading. In addition, in bypassing the normal process of tumor initiation and/or the steps required for metastasis by using cells established from metastatic sites, the process of angiogenesis and interaction with the microenvironment may not be accurately modeled. Orthotopic implantation has been suggested to represent a more accurate environment for the progression of PCa [89]. The use of orthotopic tissue implantation addresses some of the limitations listed above but does not completely recapitulate the normal processes of tumor development. However, xenograft systems have been important, and sometimes essential, to determine many physiologically pertinent results, such as the way different cells interact, or the differing tumorigenicity or metastatic capacity of specific cell populations.

#### 3.2.2. Transgenic T Antigen Models

Genetically engineered mouse models (GEMMs) have allowed for the development of mice carrying genetic modifications equivalent to those associated with human tumors. This has not only validated the importance of these alterations in tumor initiation and progression but has also allowed for the development of improved models of therapeutic efficacy for several human tumors. The first methods developed to genetically modify mice involved the introduction of DNA constructs designed to induce the expression of proteins under the control of tissue-specific promoters. This method was first used to model PCa in mice via the ectopic expression of simian virus 40 (SV40) Large T antigen (Tag) in the prostate. This approach resulted in the random integration of the transgene in the mouse genome and did not allow inactivation of gene expression. SV40 T antigens were used because of their transforming ability [90]. The large T antigen acts as an oncoprotein via suppressive interactions with the tumor suppressor proteins p53 [91] and retinoblastoma (Rb) [92] whereas the small t antigen interacts with the serine/threonine-specific protein phosphatase 2a (PP2a) [93] to induce transformation. However, because SV40 has not been observed to induce PCa in humans, the clinical relevance of the transforming actions of these antigens in mice is debatable.

**C3(1)-Tag**

The first published mouse model inducing the expression of SV40 tumor antigens to develop PCa in the mouse was the *C3(1)-Tag* model in 1994 [94]. A review written soon thereafter discussed the genetic alterations that take place during the development of prostate cancer in this first transgenic model of multi-stage PCa [95]. The researchers targeted the expression of the SV40 large tumor antigen (Tag) to the prostate by using a region of the *C3(1)* gene, which is formally known as the rat prostatic steroid binding protein gene. Male *C3(1)-Tag* mice developed prostatic epithelial hyperplasia as early as 3 months of age, and the majority of males developed locally invasive adenocarcinoma by 7–11 months of age. Tumors rarely metastasized, and those that did went to the lung. However, the expression of Tag was not specific to the prostate; two-thirds of female mice developed mammary adenocarcinoma by 12 weeks of age, and they correlated to SV40 transgene expression. SV40 expression was also detected in the salivary gland and testes. The fact that it took months for mice to develop adenocarcinoma after the first appearance of PIN suggests that genetic factors besides Tag expression, such as loss of p53, were responsible for transformation [95]. The strategy of expressing SV40 tumor antigens to the prostate using prostate-specific genes was used in several later models with more effective prostate targeting.

**TRAMP**

One of the most well-known prostate cancer mouse models is the TRAMP (transgenic adenocarcinoma of the mouse prostate) model, which was generated and characterized in 1995–1997 [96–98]. In this model, expression of both the large and small SV40 tumor antigens (T/tag) was regulated by the prostate-specific rat probasin promoter (rPB, now commonly referred to as PB). This was the first in a line of PB expression cassettes, each containing slightly different lengths of the promoter and 5′ untranslated region (UTR); this particular construct contained 426 base pairs (bp) of the PB promoter as well as 28 bp of the 5′ UTR (signified as −426/+28) [97, 99]. We will discuss the other PB expression cassettes later in the article. The success of the TRAMP model was that it was able to induce transgene expression specifically in the prostate (due to the androgen-regulated prostate-specific expression of PB) whereas previous and future models expressed the transgene in other organs.

TRAMP mice developed epithelial hyperplasia by 8 weeks of age (corresponding to sexual maturity), progressed to prostatic intraepithelial neoplasia (PIN) by 18 weeks of age, and after 28 weeks of age, 100% of the mice displayed lymphatic metastases, and approximately two-thirds displayed pulmonary metastases. Thus, TRAMP was the first mouse model to display distant organ metastases, albeit rarely to the skeleton. This model was also the first GEMM to display castration-resistant disease. Castration of mice at 12 weeks of age did not affect primary tumor development or metastasis in the majority of TRAMP mice. In mice that developed primary tumors despite castration, twice as many displayed lymphatic or pulmonary metastases relative to noncastrated mice, indicating that the mice that develop PCa after castration are predisposed to develop more aggressive and more poorly differentiated disease [96]. An issue with the TRAMP model is that the most frequent malignancy in these mice has been reported to be of neuroendocrine origin [100]. Some neuroendocrine markers are found in human PCa, but the majority of cells in prostate adenocarcinoma are epithelial. It is possible that the simultaneous loss of p53 and Rb could increase susceptibility to neuroendocrine cancer [100]. Overall, the TRAMP model may still be suitable for studying PCa, but it may only be clinically relevant for a small population of patients that develop PCa of neuroendocrine origin, or perhaps for those who have extensive neuroendocrine differentiation [101].

**FG-Tag**

The fetal globin-*γ*/T-antigen (*FG-Tag*) mouse model was originally developed (concurrently with several other less-specific mouse lines) in 1996 [102] and was further characterized a year later [103]. A molecular characterization of the *FG-Tag* model has also been published [104]. At the time, the fetal globin-*γ* gene was thought to be specifically expressed in embryonic erythroid cells based on studies in transgenic mice [105], and the authors of the study were initially interested in these erythroid cells. They generated eight mouse lines using the human *FG* promoter. In one line (referred to as G*γ*/T-15), they saw formation of palpable urogenital tumors at 16 weeks and highly vascularized prostate tumors in 50% of male mice at 5–7 months of age. Male mice also displayed metastasis to the renal lymph nodes, adrenal glands, and kidneys, along with infrequent micrometastases to the lung, bone, and thymus. Transgenic male mice were castrated at 4–6 weeks of age (sexual maturity) and prostate tumors still formed, a sign of castration resistance. In the female transgenic mice, adrenocortical tumors were seen in 50% of the mice that were surveyed, indicating that the FG promoter used to express T antigen was not prostate specific. In fact, when the model was developed, the PCa phenotype was not expected and occurred rather randomly along with phenotypes in other tissues. A followup study of the FG-Tag model found that Tag expression occurred in p63^+^ basal cells, but neuroendocrine differentiation still took place in advanced tumors [106]. Overall, this mouse line is a fairly accurate model for the study of castration resistant PCa, but its off-target effects may play a critical role in carcinogenesis.

**LADY**

The LADY series of models, developed in 1998, is similar to the TRAMP model in that the PB promoter drives SV40 T antigen expression [107], but there are two key modifications. The creators of these models recognized that while expression with the PB promoter was sufficiently prostate specific, the transgenic expression level was variable between the previously developed mouse lines. They had shown that a larger fragment of the PB promoter (LPB) could be used to cause consistently high transgene expression [108]. This LPB cassette (also known as the second generation PB promoter) contained 11,500 bp from the PB promoter and 28 bp from the 5′ UTR (−11,500/+28). This LPB promoter was subsequently utilized to express T antigen. The second key difference in this model is that the LPB promoter was linked to a deletion mutant of the SV40 T antigen that expressed only the large T antigen and not the small t antigen. Seven transgenic lines were then established and split into three groups based on the stage of neoplasia attained and how fast the cells became hyperplastic. All male mice developed prostate tumors, but the fastest line to do so was designated 12T-7f. This line was the only one to develop late-stage adenocarcinoma. The cancer started as small foci of hyperplasia, followed by proliferative overgrowth, dysplasia, HGPIN, and finally adenocarcinoma, representing an ideal model for studying the various stages of PCa. Tumors from the 12T-7f line regressed upon castration but were restored after administration of androgen. According to a later article, some mice developed metastases to the lymph nodes, liver, and lung in the 12T-10 line, but this line also showed a more neuroendocrine phenotype [109]. The LADY model moved PCa mouse modeling forward due to its more specific targeting and increased levels of transgene expression in the prostate. Also, the LADY models more accurately mimic the majority of human PCa because the cancer is slow growing and has a mostly epithelial phenotype. However, castration resistance and metastasis are not modeled well.

Because type II transmembrane serine protease hepsin mRNA has been shown to be upregulated in the majority of PCa tumors [110, 111], a PB-hepsin construct was placed with the LPB-Tag construct used in the LADY 12T-7f model such that both the large T antigen and the hepsin transgene were expressed specifically in the prostate. Laminin-332 has been shown to act as a substrate for hepsin, which may increase migratory ability [112]. This double transgenic model was developed in 2004 [113]. The PB used to express hepsin was of the third generation of PB promoters. It is known as ARR_2_PB because it comprises one copy of androgen receptor binding site 1 (ARBS-1) and ARBS-2 [114]. This cassette is composed of 286 bp of the PB promoter and 28 bp of the 5′ UTR (−286/+28) and contains the DNA sequence necessary for androgen-regulated prostate-specific expression. An added benefit of the ARR_2_PB promoter is that it consistently induces higher levels of transgene expression than its predecessors. LADY 12T-7f mice do not normally express hepsin, and the addition of hepsin expression caused disruption and disorganization of the epithelial structure, leading to invasion and distant metastasis in 55% of male mice after 21 weeks. Metastases went primarily to the liver and also to the lymph nodes and skeleton. The metastatic lesions were, however, determined to contain mainly neuroendocrine cells, which is inconsistent with human metastases. This model opened possibilities of inducing metastasis in models that normally do not show metastasis, and doing so by the expression of a gene that has been detected in human PCa (hepsin), which is ideal.

**CR2-Tag**

In 1998, the *CR2-Tag* model was developed, which placed Tag expression under the control of a segment of the cryptdin-2 (*CR2*) gene after it was discovered that *CR2-Tag* male mice of differing pedigrees were dying unexpectedly of large prostate tumors at 5–7 months of age [115]. The creators of this model were originally studying intestinal epithelial cells (Paneth cells) that secrete antimicrobial peptides called cryptdins [116]. When Tag expression was induced in Paneth cells via *CR2*, the cells died and did not cause any abnormal intestinal phenotypes in the mice; however, prostate tumors formed. By 12 weeks of age, all male mice displayed PIN, and by 24 weeks, every male mouse displayed locally invasive PCa. Mice displayed metastases to the liver, abdominal lymph node, lung, and bone marrow. These tumors were also castration resistant. However, Tag expression was once again being induced in neuroendocrine cells, which were extremely tumorigenic in this model. To further the molecular description of the prostatic neuroendocrine cells in this model, laser microdissection was later performed for functional genomics analysis [117].

**PSP94-Tag**

To establish an additional vector gene system for the study of PCa, the *PSP94-Tag* model (known as TGMAP, for transgenic mouse adenocarcinoma in the prostate) was developed in 2002 [118]. PSP94 refers to the prostate secretory protein of 94 amino acids, which is one of the three most abundant secretory proteins in the prostate. PSP94 was used to express both large and small T antigens in the prostate. Three founder lines were established (183-2, F0183-3, and F0186-9). Line 183-2 was similar to the TRAMP model, while the other two were similar to the LADY model. Metastatic PCa was found in some of these mice in the lymph node and kidney. Some tumors regressed after castration, but a population of mice was eventually castration resistant. However, the TGMAP model showed variable phenotypes and genomic variability. Therefore, in 2005, a new PSP94-Tag model was created using a novel gene-targeting method; it was called the knockin mouse adenocarcinoma of the prostate (KIMAP) model [119]. KIMAP was a better model in that all male mice developed slowly progressing PCa synchronously, there was a higher tumor penetrance, neuroendocrine differentiation was rarely seen, and it mimicked human disease in other aspects [120]. The KIMAP model is the most comparable to human disease out of the models expressing SV40 Tag, especially because of its relative lack of neuroendocrine differentiation.

#### 3.2.3. Other Transgenic Models

**Mt-PRL**

*Mt-PRL* was created in 1997 to determine the physiological role of the polypeptide prolactin (PRL) in the prostate gland [121]. PRL levels increase with age and during prostate hyperplasia [121]. The prostate also expresses PRL receptor, and various effects have been observed in the prostate upon PRL activation [122]. Therefore, the contribution of PRL expression to PCa is unclear. The metallothionein-1 (Mt-1) promoter was placed upstream of the *PRL* gene to induce its expression. Mt-1 expression is not restricted to the prostate, however, meaning that expression of *PRL* was being directed to any tissue that naturally expresses Mt-1 [123]. *PRL* transgene expression was detected in the liver, thymus, kidney, pancreas, seminal vesicles, testes, and prostate. The authors knew that PRL was important in mammary gland development, but they wished to determine the role of PRL in the prostate [121]. All prostates examined were enlarged, and all were hyperplastic with increased stroma as well as nuclear polymorphism. Testosterone levels were increased in *Mt-PRL* mice. No metastasis or castration resistance was reported. This model would be more appropriate for benign prostatic hyperplasia (BPH) than for PCa. To more accurately elucidate the mechanism of PRL in PCa, a more prostate-specific promoter should be used in future models.

**BK5-IGF1**

Soon after the LADY and *CR2-Tag* models were being developed, a transgenic mouse model was created that overexpressed the insulin-like growth factor 1 (IGF1) under control of the bovine keratin 5 (BK5) promoter [124, 125]. It had been shown that elevated IGF1 expression was an indicator of PCa, even in some cases in which PSA levels are normal [126]. The creators of this model originally chose the BK5 promoter to induce IGF1 expression in order to study the specific role of IGF1 in carcinogenesis in the basal layer of the epidermis. BK5 is expressed in the basal epithelial layer of multiple tissues, including (but not specific to) the prostate. They found prostatic hyperplasia in mice as young as 2 months. After 6 months, mice displayed hyperplasia, dysplasia, and PIN in all prostatic lobes and adenocarcinoma after 9 months. Some neuroendocrine differentiation was detected. The prostate tumor cells were locally invasive but did not metastasize nor were they castration resistant. Similar to the *Mt-PRL* model, the *BK5-IGF1* model may be adequate to model prostate disease, but the off-target effects of the transgene would be cause for concern when interpreting results.

**PB-mAR**

The purpose of the *PB-mAR* model, developed in 2001, was to determine the direct effect of the androgen receptor (AR) on the prostate epithelium [127]. This may seem unusual, considering that it was already well known that the majority of PCa cases are androgen dependent for tumor growth and indeed express AR, and drugs were already being manufactured to ablate AR activity [128]. However, before this, no animal model had been created to directly test the effects of AR overexpression. Murine AR (mAR) expression was placed under control of the first generation PB promoter (−426/+28). All male mice displayed hyperproliferation, neoplasias, and eventually microinvasive HGPIN, indicating that AR is a positive regulator of cellular proliferation and that mouse prostates are more susceptible to PCa upon AR overexpression. However, a more recent finding suggests that the loss of AR expression in the mouse prostate via PB-Cre recombination also results in increased epithelial proliferation [129]. Therefore, more needs to be learned about the mechanism of how AR behaves in the prostate. This model would be valuable in future AR studies, especially in terms of castration resistance.

**ARR_2_PB-Myc**

The functional role of Myc in PCa is unknown, but it has been found to have an increased gene copy number in approximately 30% of human PCa cases [130, 131]. Therefore, to determine the effect Myc has in the prostate, a model was generated in 2003 [132]. The PB promoter (−426/+28) was used to generate mice with low Myc expression (“lo-Myc”), and the ARR_2_PB promoter was used to generate mice with high Myc expression (“hi-Myc”). The hi-Myc mice displayed progression from PIN to invasive carcinoma as early as 3 months whereas the lo-Myc mice displayed the transition at 10 months, indicating a dosage-dependent response to the *Myc* transgene. This data also implies that the two PB promoters have differing ability to induce transgene expression. Upon castration at 2 months, all mice showed complete regression of PIN. When mice with PCa were castrated at 8 months, tumors regressed, but the mice displayed residual tumors up to 5 months after castration. Thus, it seems that early in the progression of PCa, androgen ablation is fully effective, but after the cancer has had time to develop, androgen ablation is less effective. No metastasis was observed in this model, so ARR_2_PB-hepsin transgenic expression was added to the Myc model by crossing PB-hi-Myc to PB-hepsin mice to determine if metastases would result (this was the same strategy used in the LADY model) [133]. These bigenic mice displayed higher grade carcinoma and swifter tumor progression but did not develop any metastases. All of the Myc models also developed adenocarcinoma, rather than neuroendocrine carcinoma. These models would be most practical for studying early progression of PCa and stand to contribute to Myc research in PCa.

**ARR_2_PB-FGFR1**

In 2003, the physiological roles of fibroblast growth factor receptors 1 and 2 (FGFR1 and 2) in the prostate were determined [134]. FGFR1 had been shown to be upregulated in 40% of poorly differentiated PCa [135]. The founder lines were called JOCK1 and JOCK2, respectively. What separates these models from others is that they used chemically induced dimerization (CID) technology to regulate FGFR signaling. Treatment with AP20187 induced signaling in the mice, and withholding treatment stopped signaling. FGFR2 signaling did not have an effect whereas FGFR1 signaling induced pronounced hyperproliferation and PIN, which was reversible until neovascularization occurred. This indicates the importance of FGF signaling in PCa. Two downstream targets of FGFR1 (ERK1 and ERK2) were activated via phosphorylation and translocated to the nucleus in regions of hyperplasia and progression. The *PB-FGFR1* model represents a good model for early stages of PCa but fails to recapitulate more aggressive aspects of the disease. Crossing mice from this model to other mice may induce metastasis and provide information about possible synergism between the FGF pathway and other molecular signaling pathways.

**PB-Ras**

*PB-Ras* mice were generated in 2004 to test the effects of oncogenic H-Ras on the prostate [136]. Ras has been implicated in many cancers and works through many downstream effectors (including the MAP kinase pathway) to induce proliferation, cell survival, and growth. A mutation at residue 12 (Ras^val12^) changing the amino acid to a valine causes Ras to become constitutively active, and expression of this version of *Ras* was placed downstream of PB (−426/+28). Activated Ras has been detected in human PCa, but it has been suggested that Ras mutations are rare and therefore insignificant in PCa [137–139]. Male mice did not progress further than PIN. Other phenotypes included intestinal metaplasia and thickened fibromuscular stroma. This model, similar to the *PB-FGFR1* model, indicates that Ras may be important in early PCa transformation but may not be as important in later stages.

**PB-Neu**

Neu (also known as ERBB2 or HER2) has been implicated in various cancers and is best known for its strong correlation with a subtype of breast cancer [140]. In 2006, a novel PB promoter was utilized to induce expression of a constitutively active version of *Neu*; the PB used was 4,500 bp [141]. All sacrificed male mice displayed at least one of three prostate phenotypes: hyperplasia, PIN, or PCa, depending on the age at sacrifice. Only 7 of 60 (11.7%) mice developed PCa. Of the mice that developed PCa, five displayed considerably enlarged prostates that included recruitment of fibroblasts and angiogenesis, but no invasion or metastasis was apparent. Histopathologically, the carcinoma that developed in this model is similar to the acinar type in humans that is believed to be derived from luminal epithelial cells. Neu expression in human PCa has been detected, but the frequency of samples that express Neu is debatable and has been reported anywhere from 10 to 41% of cases [141]. This model shows that Neu activation can induce PCa in mice. Therefore, *PB-Neu* could serve as a valuable model for elucidating the tumorigenic signaling through Neu, but it remains to be seen whether it will have physiological significance in human PCa.

**ARR_2_PB-ERG**

The fusion between transmembrane protease, serine 2 (TMPRSS2) and ETS-related protein (ERG) is the most common fusion that takes place in the *ERG* gene. The TMPRSS2-ERG fusion protein is positively expressed in approximately 50% of human PCa cases [142]. When the genes become fused, the noncoding exon 1 of *TMPRSS2* is placed next to exon 2 of *ERG*, resulting in a truncated *ERG* product. In 2008, a mouse model was created that placed this *ERG* product under control of the ARR_2_PB promoter [142]. The mice developed PIN by 12–14 weeks of age at a frequency of about 38%. This suggests that the TMPRSS2-ERG fusion protein is not sufficient to induce PCa, but this model must be evaluated at later timepoints to validate this assertion. Other groups have also generated transgenic ERG models with mixed results [143–145]. Only a small percentage of the mice developed low grade PIN, and most mice did not develop PIN; the authors suggest that the interpretation of the histology may be the reason for varying results and that the PIN in the *ARR_2_PB-ERG* model was rather subtle. A novel finding that came out of these studies was that concomitant TMPRSS2-ERG expression and loss of PTEN or activation of the PI3K pathway results in more aggressive PIN that appears at an earlier age. Carver et al. showed that transgenic ERG expression coupled with heterozygous Pten expression results in invasive prostate adenocarcinoma in mice [143]. Taking these results together, it appears that ERG fusion proteins have a subtle role in prostate tumor initiation and may cooperate with the PI3K pathway to induce tumorigenesis, but more needs to be done to fully elucidate the molecular mechanisms involved.

**Summary 3.2.3**

All the transgenic models discussed thus far show an interesting phenomenon: while SV40 T antigen is not normally expressed in human PCa, its induced expression generally causes particularly aggressive, metastatic, and castration resistant PCa (Table 1). However, when other oncogenes (which may or may not be expressed in human PCa cases) are expressed specifically in the prostate, the resulting phenotype is mild and rarely progresses to adenocarcinoma (Table 2). This is most likely due to the T antigen's negative effects on p53 and Rb (which is generally only seen in late disease in humans), which also probably accounts for the neuroendocrine phenotype. Transgenic models have so far not been able to accurately induce all stages of epithelial PCa in a mouse using an endogenously expressed gene. This is most likely because PCa probably requires more than one genetic event involving multiple molecular pathways. There is much work to be done to interpret and validate the findings from this multitude of transgenic mouse models.

#### 3.2.4. Traditional Knockout Models

Traditional (or whole-body) knockout models represent a different strategy for determining the roles of important genes in PCa. Tumor suppressor genes (or regions of DNA essential for their activity) are excised in knockout models. Historically, these models were valuable in determining embryonically lethal genes. If, after a gene was knocked out, the embryo did not survive, the gene was considered embryonically lethal and therefore critical in development; however, embryonic lethality precluded the widespread use of knockout models for cancer. When a traditional knockout is created, the gene of interest is knocked out ubiquitously, so it is difficult to determine the organ-specific roles of that gene. Traditional knockouts are generally made via homologous recombination in embryonic stem cells (ESCs). This process was first described in 1989, and the developments leading to this were recognized with the 2007 Nobel Prize in Physiology or Medicine [146]. Readers will notice that these knockout models, even when PCa is observed, have generally not been tested for castration resistance. For complete analysis of these models, such tests should be performed.

**RAR*γ***

The retinoic acid receptor gamma (RAR*γ*) was knocked out to make a mouse model in 1993 [147]. The purpose of the model was to determine unique functional roles of RAR*γ* receptors, with little thought to prostate phenotypes (although the RAR family had been suggested to be important in organogenesis). RAR*γ* family member expression had been shown via *in situ* hybridization to be limited to several tissues in the embryo and in the skin of the adult. A study from 2000 showed that various RARs (including gamma) are expressed in a majority of the PCa samples that were tested [148]. Eight out of 8 male mice developed squamous cell metaplasia in the prostate and seminal vesicles and were unable to impregnate female mice. There were also developmental issues in the prostate, evident in the lack of normal mucosal folds, septa characteristics, and secretory products. A vast array of phenotypes was observed in these mice, but carcinoma was not detected, and metastasis and androgen dependence were not checked. This is not a good model for PCa.

**p27**

Three groups simultaneously published the generation of a *p27* (also known as Kip1) knockout mouse in 1996, but they did not include analysis of prostatic tissue [149–151]. p27 is a tumor suppressor that plays a role in G_1_ phase arrest during the cell cycle. In 1998 this model was examined for effects on the prostate because distinct p27 expression patterns had been identified in human PCa samples, and loss of p27 seemed to correlate to more aggressive PCa [152]. *p27* knockout mice display hyperplasia of multiple organs, aside from other phenotypes (Table 3). Prostatic hyperplasia was detected in acinar epithelial cells upon histological analysis, similar to that seen in human BPH. Increased proliferation, enlargement of the glands, and increased fibromuscular stromal cells were observed, but metastasis and androgen dependence was not tested. The *p27* mouse may not represent a critically important model for PCa but may be a good model for BPH, as it seems that loss of p27 is causally linked to BPH development in both humans and mice [152].

**Pten**

Pten, or “phosphatase and tensin homolog deleted from chromosome 10” (also known as Mmac1, for mutated in multiple advanced cancers), is a key tumor suppressor, and its loss has been linked to many cancers, including a strong correlation in human PCa [166]. Pten is a phosphatase that removes a phosphate group from phosphatidylinositol 3,4,5-triphosphate (PIP_3,4,5_), thereby downregulating the Akt-mTOR signaling pathway. In 1998, a *Pten* knockout mouse was created by generating a null mutation in the *Pten* gene [153]. Another group (in 1999) also generated a *Pten* knockout mouse by deleting a slightly different portion of the gene but inducing the same inactivating effect on Pten [154]. It was found that Pten is essential for early embryonic development, as no homozygous knockouts were viable. Heterozygotes, however, were viable and produced a broad range of phenotypes in multiple tissues (Table 3). Both models were reported to have enlarged, complex, hyperplastic prostates, and the model from 1999 reported the development of PIN. No metastases were reported, and due to the diffuse nature of the effects of Pten inactivation, castration resistance was not tested. These models showed that Pten is a critical early regulator of PCa development.

At least three other models have since knocked out an additional gene simultaneously with the *Pten* gene in what are referred to as double knockouts, in which loss of Pten with other tumor suppressors leads to more aggressive phenotypes [162, 165, 167]. The first model knocked out both Pten and p27, and 100% of male mice developed PCa, leading to the conclusion that Pten and p27 have cooperative roles in the prostate [162]. The second model knocked out both Pten and Nkx3.1, and extensive hyperproliferative multifocal lesions were observed, indicating the presence of HGPIN and adenocarcinoma, which was not observed in either the *Pten* or *Nkx3.1* models. Also, lymph node metastasis and castration resistance were reported in this double knockout [163, 164]. No increase in low-grade PIN was observed, indicating that the cooperativity of Pten and Nkx3.1 occurs at later stages of PCa [167]. Finally, when Pten and p53 (a tumor suppressor that induces apoptosis after cellular damage) were knocked out together, PIN developed with a shortened latency, indicating these two tumor suppressors cooperate to accelerate tumorigenesis [165]. Each of these models displayed epithelial carcinogenesis. Overall, Pten loss plays an integral role in the development of PCa, and the models in which Pten is lost will be of great clinical value for patients with Pten mutations.

**Nkx3.1**

Nkx3.1 is a transcription factor with tumor suppressing activity and is the murine homolog of the *Drosophila* gene *bagpipe*. Loss of Nkx3.1 is seen often in the early stages of PCa in humans [168]. In 1999, an *Nkx3.1* knockout mouse was generated to determine its physiological effects [155]. Several phenotypes were seen in the prostate and seminal vesicles. Prostates developed hyperplasia and dysplasia that advanced with age, but no overt tumors were detected. Prostate epithelial cells were hyperproliferative. Heterozygous Nkx3.1 expression resulted in a less severe form of PIN, indicating Nkx3.1 haploinsufficiency. Upon further examination of an *Nkx3.1* knockout model, morphogenic defects in the salivary glands were also found [156], indicating that Nkx3.1 may be important in the development of other cell types. The data suggests that Nkx3.1 loss is important for initiating events in PCa but may not be sufficient for progression to advanced stages; this evidence was supported in a later publication [157]. An interesting observation is that loss of Nkx3.1 disrupts normal prostatic development and differentiation, but loss of Nkx3.1 also contributes to PCa. New evidence suggests that loss of Nkx3.1 increases DNA damage, such as mutations, thereby creating an environment for genomic instability and tumorigenesis [169]. Because Nkx3.1 is downregulated in humans, Nkx3.1 mutant models are valuable in studying PCa progression.

**Stat5a**

Stat5a is a transcription factor that mediates signaling through PRL to influence the development of certain glands (see the *Mt-PRL* Section 3.2.3 for more discussion). A *Stat5a*-null mouse was generated in 1997, and Stat5a was shown to be critical in mammary gland formation and lactogenesis [158]. In 2000, Stat5a was also implicated in prostatic epithelial growth via PRL and was shown to be associated with more aggressive PCa in human cases [159, 160]. Therefore, the *Stat5a* mouse was used to determine its effect on prostate growth and development [161]. *Stat5a* mice showed a specific epithelial defect in the prostate, apparent by acinar disorganization and development of cysts. They also found that testosterone serum levels were normal in knockout mice, indicating that regardless of androgen status, Stat5a deficiency will cause developmental defects. The strategy behind this model is not one we have seen in previous models. Generally, when a gene is knocked out, it is to test its tumor suppressive activity, so that researchers can generate a cancer model. In this model, a putative oncogene was knocked out to determine if it is important in the growth and development of the gland. Later, however, researchers determined *in vitro* and *in vivo* that Stat5 is indeed an important factor in PCa metastases [170], though no prostate-specific transgenic *Stat5a* mouse has been created.

**Summary 3.2.4**

Traditional knockouts have been important to illustrate the role that certain tumor suppressors play in the prostate, particularly in early transformation. Similar to the transgenic models, the knockouts suggest that loss of any one gene is not sufficient to result in PCa and that multiple genetics events are required. However, because these models employ a whole-body knockout, it is difficult to conclude that any of these genes play a prostate-specific role due to the wide range of physiological responses that might take place in other tissues or cells that may affect carcinogenic transformation. Also, a whole-body knockout does not accurately recapitulate what generally happens in a human cancer (unless a mutation is inherited through the germline). It is commonly believed that genes are mutated or lost in one cell, allowing it to obtain “hallmarks of cancer,” [171] thereby eventually creating a tumor. A final issue with the traditional knockout is that if a gene is required for normal development of the organism, knocking it out will result in embryonic lethality, which does not allow for further study. Therefore, a more tissue-specific gene knockout model may yield more interesting and physiologically relevant results.

#### 3.2.5. Conditional Knockout Models

**The Cre-loxP System**

The Cre-loxP system was developed in bacteriophage in Sternberg's laboratory almost 30 years ago [172, 173]. “Cre” is a gene that “causes recombination,” and “loxP” is a 34-base pair “locus of phage crossing over” [173]. Cre is a recombinase protein that promotes specific genetic recombination *in trans* at loxP sites. At first, the Cre-loxP system was characterized via isolation of DNA from plaque-forming P1 phage that was able to infect *Escherichia coli*. Eventually, the Cre-loxP system was used for genetic recombination in eukaryotic cells, first in yeast and later in mice [174]. The system was further revised so that Cre could be fused to a mutated version of the estrogen receptor, which allowed Cre recombination to be dependent on tamoxifen treatment, rather than on estradiol [175].

**Figure 4:**
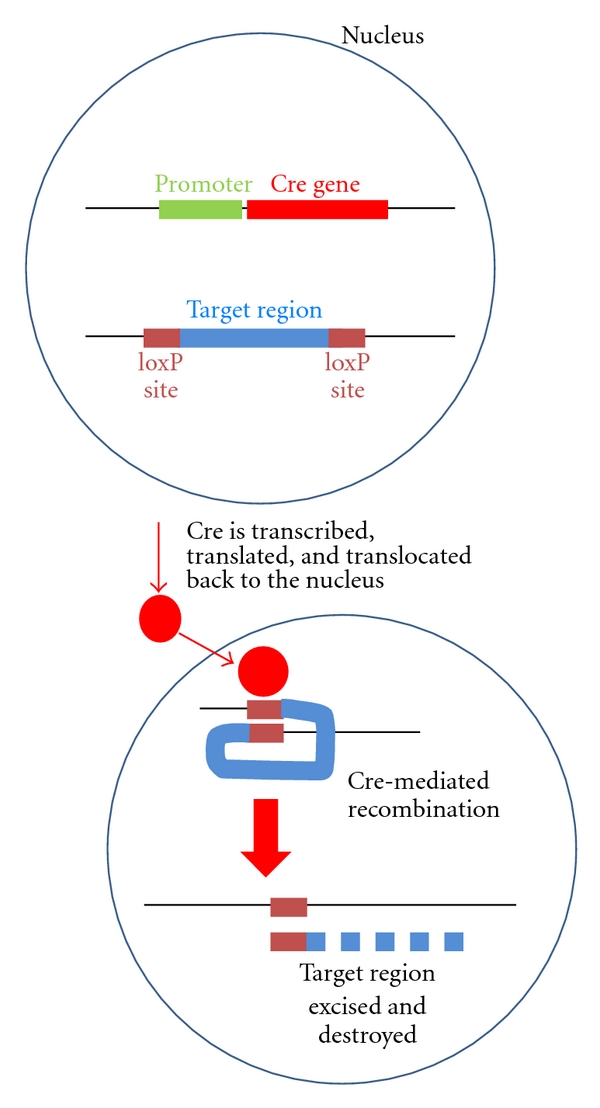
Schematic diagram of the Cre-loxP system.

The Cre-loxP system is now used extensively in mouse models for cell type-specific and tissue-specific genetic alterations. Mice that express the Cre recombinase under the control of a tissue-specific promoter are crossed with mice that express a nonspecific “floxed” genetic region, meaning that the region is flanked by loxP sites (Figure 4). Cre cuts through the loxP sites and excises any region of DNA in between, leaving behind a single loxP site. This specific recombination can either activate or inactivate a gene, depending on what genetic region is being removed. An essential exon for a gene's activity may be removed, rendering the gene inactive or “conditionally knocked out.” Alternatively, signals that prevent gene expression (a “stop” site) may be removed upstream of a mutated gene, causing the transcription of a constitutively active form of a protein. Examples of these will be discussed in subsequent paragraphs. Conditional targeting of genes avoids off-target effects and developmental issues, and it allows for both alleles of the genes of interest to be knocked out without the problem of embryonic lethality. Another added benefit to conditional models over other GEMMs is that they are able to add precision that was previously unattainable, especially in the transgenic models which randomly integrated into the genome. Due to the large number of conditional knockout models, we will discuss the different types of prostate-specific Cre that have been used to generate models and then discuss some of the individual models that have been created. Note that all of the conditional knockout models we discuss have been generated in the last 10 years (Table 4).

**PSA-Cre**

Prostate-specific antigen (PSA) is a serine protease that is almost exclusively expressed in prostatic luminal epithelial cells in the human prostate [191]. PSA serum levels are used to clinically diagnose PCa. These facts make the *PSA* promoter a strong candidate to induce targeted Cre expression in the prostate. *PSA-Cre* mice may be mated with any mouse having a floxed genetic region to induce genetic deficiency of that region. *PSA-Cre* mice have been used to conditionally knock out Nkx3.1 expression (*PSA^Cre^Nkx3.1^flox^*) [176] and Pten expression (*PSA^Cre^Pten^flox^*) [177]. PSA-Cre activity was observed in all prostate lobes but in no other tissue tested. Mice in the *PSA^Cre^Nkx3.1^flox^* model displayed hyperproliferation, hyperplasia, and PIN, which supports the idea that Nkx3.1 is important in early stages of PCa, but perhaps not later stages. Whereas the ubiquitous *Pten* knockout models reported PIN as the most severe effect observed, the conditional *Pten* knockout model reported hyperplasia, PIN, invasive carcinoma, and infrequent lymph node metastases. The results obtained from these models may more accurately represent the physiological roles of the respective genes in the prostate and, therefore, provide stronger evidence for the clinical relevance of these genes in PCa. These models display epithelial carcinogenesis, and because these genes are commonly mutated or lost in human cancers, these models provide a stable platform for the study of PCa progression. The lack of skeletal metastases, however, limits these particular models to study of earlier stages of PCa.

**Probasin-Cre**

The PB promoter was extensively used in the SV40 T antigen models and has been shown to be prostate-specific on numerous occasions. We have discussed the first, second, and third generations of the PB promoter. Two version of Probasin-Cre have been generated, one using the −426/+28 PB, known as PB-Cre [192], and the other using the ARR_2_PB, commonly known as PB-Cre4 (the specificity of PB-Cre4 to prostate epithelial cells avoids the neuroendocrine phenotype) [193]. We will collectively refer to them as Probasin-Cre, but will specifically use the PB-Cre and PB-Cre4 terminology for individual models. Probasin-Cre has been the most widely used promoter in PCa conditional knockout models. Genes that have been knocked out using Probasin-Cre include *Pten* [178, 179], *Rb* [180, 181], *p53* [180, 181], *Apc* [183], *IGF-1* [184], and *Brca2* [187]. Probasin-Cre has also been used to overexpress certain oncogenes by excising a STOP codon upstream of the gene, such as with *Kras* [185], *Catnb* [185, 186], and *Alox15* [182]. We will discuss a few of these models here; see the original articles for more detail (Table 4).

Two models were created knocking out the Rb tumor suppressor in the prostate [180, 181]. When *Rb* was knocked out alone (*PB^Cre^Rb^flox^*), the most severe prostate phenotype observed was hyperplasia with loss of integrity of the basement membrane, suggestive of preinvasive lesions [180]. The conclusion was that Rb loss alone was not sufficient to cause transformation. A similar conclusion was drawn from a conditional *p53* knockout [181]. When Rb loss and p53 loss were coupled (*PB^Cre4^Rb^flox^p53^flox^*), metastatic carcinoma resulted [181]. Prostate tumor tissue invaded surrounding tissues, including adipose tissue, muscle, blood vessels, bladder, and urethra, and distant metastases were found in the lungs, liver, adrenal gland, and lymph nodes. These results indicate that p53 and Rb cooperate in the prostate during tumor progression and metastasis. *PB^Cre4^Rb^flox^p53^flox^* is similar to TRAMP in its aggressiveness, which makes sense because the large T antigen has deleterious effects on both p53 and Rb. A basal phenotype was lost in this model, but luminal markers and AR were expressed. Neuroendocrine differentiation was also detected, especially in metastatic lesions and after castration resistance occurred. This histopathology is similar to what is seen in humans, and different from TRAMP because that model did not display adenocarcinoma. Therefore, *PB^Cre4^Rb^flox^p53^flox^* represents a valuable model for studying multiple stages of PCa but falls short in terms of skeletal metastasis.

The *Pten* gene is one of the most studied genes in PCa, and it was knocked out conditionally in mice (*PB^Cre^Pten^flox^* and *PB^Cre4^Pten^flox^*) [178]. These models again point to the importance of Pten in the prostate, and it was shown that the level of Pten knockdown dictates the progression of PCa. Loss of heterozygosity of Pten led to a shortened latency of PIN formation. The conditional inactivation of Pten had complete penetrance, and the prostate tumors were invasive and diffuse. Another article was published at the same time that showed that *PB^Cre4^Pten^flox^* mice developed metastasis in the lymph nodes and lung [179]. Notably, this was the first mouse model in which deletion of an endogenous gene induced metastatic PCa. Castration resistance was detected, meaning that this model represents one of the most accurate models of PCa yet created, especially because Pten is frequently lost in human PCa [194]. Although tumors in these mice do not appear to metastasize to the skeleton, *PB^Cre4^Pten^flox^* is a useful model for the study and characterization of PCa.

The canonical Wnt/*β*-catenin signaling pathway may play an important role in PCa development. To investigate the interaction of Wnt signaling in the prostate, a model was generated to knock out the adenomatous polyposis coli (*APC*) gene, which is a negative regulator of the Wnt/*β*-catenin pathway [183]. By knocking out *APC*, the Wnt pathway is constitutively active. *PB^Cre4^Apc^flox^* mice developed PIN followed by locally invasive adenocarcinoma, but did not display distant metastasis. Upon castration before tumorigenesis, carcinoma did not develop, but castration after tumorigenesis resulted in retention of adenocarcinoma. This indicates that these tumors are dependent on androgens for tumor initiation but are castration resistant during tumor maintenance and progression. Similar results were seen in the *PB^Cre4^Catnb^lox(ex3)^* mouse model, in which *β*-catenin was overexpressed in its constitutively active form [185]. Due to the accumulating evidence that the Wnt/*β*-catenin pathway is upregulated in PCa, this model proves to be valuable.

**MMTV-Cre**

The mouse mammary tumor virus (MMTV) has also been used to induce Cre expression. This is one of the weaker constructs used to express Cre in conditional models, due to its expression in many tissues including prostate, mammary, skin, salivary gland, and seminal vesicles. Therefore, any floxed region that is present in those tissues during Cre recombination will be excised and could develop a unique phenotype that affects PCa development. In one model (*MMTV^Cre^Catnb^lox(ex3)^*), MMTV-Cre was used to remove exon 3 (which stabilizes the *β*-catenin protein) from the *β*-catenin (*Catnb*) gene, thereby activating the Wnt signaling pathway [188]. As expected, this model displayed phenotypes in various tissues, including skin and mammary. In the prostate, stabilization of *β*-catenin caused hyperproliferation and loss of differentiation, resulting in hyperplasia and metaplasia but not carcinoma. Another model (*MMTV^Cre^Pten^flox^*) knocked out *Pten* in the prostate. Cre-mediated recombination was also detected in several other tissues due to the range of MMTV expression [189]. The prostates of these mice displayed increased proliferation and HGPIN but did not progress to carcinoma or castration resistance. It is difficult to make salient conclusions about these models due to the multiple off-target effects. Therefore, these models are not as valuable as those that are prostate specific.

**FSP1-Cre**

The *FSP1^Cre^TGF*β*^flox^* model places Cre expression under the control of the fibroblast-specific protein 1 (*FSP1*) gene, which is exclusively expressed in fibroblasts after day 8.5 of embryonic development [190]. The inactivated gene in this model is transforming growth factor *β* (*TGF*β**), which is an important signaling molecule in epithelial and mesenchymal interactions and may play a role in neoplastic transformation of epithelial cells. Cre recombination occurred in many other tissues because fibroblasts are present throughout the body. PIN was seen in the prostates of these mice, while many other tissues were histologically normal. This indicates that TGF*β* may play a tumor suppressive role via fibroblast secretion in the prostate. This model may be important in elucidating a role for TGF*β* in tissue differentiation, but it is not specific to the prostate.

**Nkx3.1-Cre^ERT2^**

A recently described addition to the inventory of prostate-specific Cre-expressing strains is the *Nkx3.1-Cre* mouse that has thus far been used in one published conditional model (*Nkx3.1^CreERT2^Pten^flox^*) [16]. The homeobox protein Nkx3.1 has been shown to be expressed on a rare population of luminal epithelial stem cells called castration resistant Nkx3.1-expressing cells (CARNs) [16]. The Cre used in this model was fused to an ERT (tamoxifen-inducible estrogen receptor) vector so that Cre is only active upon tamoxifen treatment. Therefore, Cre activity can be controlled so that the floxed region can be excised at specific time points. It is important to note that tamoxifen treatment could have phenotypic effects on the prostate (such as lowering testosterone levels [195]); therefore, experiments must be thoroughly controlled. An additional stipulation is that insertion of Cre results in one null allele for *Nkx3.1*, making its expression heterozygous. This is important because Nkx3.1 is haploinsufficient: the loss of one *Nkx3.1* allele results in mild PIN, and this could contribute to any phenotype seen in these conditional knockouts. The *Nkx3.1-Cre^ERT2^* mouse was crossed to the *Pten-flox* mouse to knock out Pten activity in the prostate. This resulted in rapid formation of hyperproliferative PIN and microinvasive carcinoma, indicating that CARNs can act as a cell of origin for prostate cancer, which has widespread implications for PCa research as a whole. This model has clinical relevance because if a castration resistant luminal stem cell is a cell of origin for PCa, then it is possible that the castration resistance seen in aggressive PCa is a result of transformation of this cell type [16]. This could lead to targeted therapy for castration resistant PCa. The use of *Nkx3.1^CreERT2^* to inactivate other genes will be of great interest in future models.

**Summary 3.2.5**

The conditional models we have discussed have mostly knocked out the same genes that were knocked out using the whole-body strategy. There are several advantages of the conditional models over the traditional models. The genetic event in the conditional models is localized to the prostate, thereby allowing a more focused study of the genes' roles. The conditional model allows for the study of genes whose loss results in embryonic lethality, such as *Rb* or *p53*, because those genes are still being expressed in every other tissue. Conditional models are more accurate in terms of mimicking the genetic events in a majority of cancer patients in that a small portion of the body (even specific cell types) display the genetic event, as opposed to the entire organism. Finally, conditional models can be temporally controlled, for instance through tamoxifen-inducible systems. The prostate phenotypes seen in the conditional models are often more severe than those seen in the traditional models. Consider the *Pten* knockouts in particular; the traditional *Pten* models resulted in PIN, but the conditional models resulted in adenocarcinoma and metastasis (Tables 3 and 4), which may be a more accurate representation of what happens in human patients. These disparate results indicate that there is still more to learn about the tumorigenic properties of these genes in the prostate.

#### 3.2.6. Current and Future Models

No current mouse model fully recapitulates all features of PCa. Xenograft studies can swiftly add insight into molecular mechanisms and microenvironment factors important in PCa. Years of mouse modeling have also shown that mice can be predisposed to cancer following correct manipulation of the germline. The development of the Cre-loxP system has opened doors to the potential generation of numerous PCa mouse models; its flexibility and utility will allow for swift development of newer and more accurate models. A common strategy for developing new models is to determine commonly deregulated genes in human PCa (via microarray analysis, for instance) and then induce overexpression or deletion of those genes in mice. There are several articles that discuss the various genetic abnormalities—including mutations, insertions and deletions, and chromosomal translocations—that exist in PCa [2, 196–200]. Theoretically, any of these genes can be expressed or deleted to determine their physiological role in tumorigenesis in the mouse. There are still many untested genes. 

An important point to make about GEMMs models is that the mutation, excision, or overexpression of the gene in question occurs throughout the entire body (in the transgenic or whole knockouts) or entire tissue (in the conditional models), and these genetic changes are present from early development or birth. This does not compare well to the situation in humans, in which mutations often occur at random, possibly in a single cell, resulting in focal disease development. It would be shortsighted to limit the scope of the animal model to the types of models described in this paper. We can expect newer, more sophisticated, and more biologically diverse models in the future, which will ideally address the challenging issues of the cell of origin for PCa, new targets for castration resistant PCa, and the molecular mechanisms and crosstalk causing skeletal metastasis. A relatively unexplored area of PCa research in mouse modeling is the role of the immune system, such as inflammation or regulatory T cells, on PCa initiation, progression, and metastasis. It is becoming more widely accepted that the immune microenvironment, not only the deregulation of genetic pathways, is important in PCa. While a wealth of knowledge has been gained thus far from mouse models, there is a need to come to a common ground on what is agreed upon in the field and what remains to be discovered [49].

## 4. Conclusions

Ultimately, the goal of developing accurate experimental models in the mouse is to study the effects of drugs for treating human disease. Especially imperative in PCa research is the search for preventative measures or therapies for castration resistant disease and skeletal metastases. Models that accurately recapitulate human disease can also be used to determine the importance of various environmental influences such as vitamin D, diet, and other factors; some of these studies have already been done with several of the models we have discussed [201, 202]. The development of mouse models has brought us ever closer to finding better therapies for PCa. 

Mouse models are suited to answering important questions in PCa research, such as the cell of origin for PCa, molecular differences between indolent and aggressive PCa, and factors that are necessary for metastasis of PCa tumor cells to the skeleton. However, mice may not display all the heterogeneity seen in humans or represent every stage of human PCa, and their tumors may not metastasize to the same tissues as in humans. All of these possibilities must be taken into account when developing a mouse model, and the best way to accurately represent all of these aspects is to target the correct genes in the correct cell(s) of origin of PCa. Mouse models as a whole represent rich heterogeneity of genetic expression. One model will probably not fully encompass all of the various stages and aspects of human disease. Instead, a variety of models may be needed to study different aspects of PCa, and xenograft models may likely play a critical role, especially in metastatic studies. In order to expand the present collection of models, we must know the genetic variations and gene expression patterns so that we can accurately categorize PCa. The pairing of genetic characterization of human PCa and the improvement of mouse models will be the most effective way of advancing overall knowledge and available treatments for this widespread disease.

## Figures and Tables

**Table 1:** Prostate cancer mouse models utilizing T antigen.

Model	Year	Other tissues affected	Metastasis sites	Cell of origin	Castration resistance	References
*C3(1)-Tag*	1994	Mammary gland, testes, salivary gland	Infrequently to the lung	Epithelial	Not determined	[94, 95]
*PB-T/tag *(TRAMP)	1995	None	LN, lung	Neuroendocrine	Yes	[96–100]
*FG-Tag*	1996	Adrenal gland, adipose tissue	LN, adrenal glands, kidneys	Neuroendocrine	Yes	[102–104, 106]
*LPB-Tag *(LADY)	1998	None	LN, liver, lung	Neuroendocrine	No	[107–109]
*CR2-Tag*	1998	Paneth (intestinal epithelial) cells	LN, lung, liver, bone marrow	Neuroendocrine	Yes	[115–117]
*PSP94-T/tag (TGMAP & KIMAP)*	2002	None	LN, kidney	Epithelial & rarely neuroendocrine	Yes	[118–120]
*LPB*-*Tag/ARR_2_ PB-hepsin *	2004	None	Lung, liver, bone	Neuroendocrine	Not determined	[113]

**Table 2:** Prostate cancer transgenic mouse models.

Model	Year	Other tissues affected	Highest stage of neoplasia	Castration resistance	References
*Mt-PRL*	1997	Liver, thymus, kidney, pancreas, seminal vesicles, testes	Benign hyperplasia	No	[121–123]
*BK5-IGF1*	2000	Basal epithelial cells	Adenocarcinoma	No	[124, 125]
*PB-mAR*	2001	None	HGPIN	Not determined	[127]
*ARR_2_PB-myc*	2003	None	Adenocarcinoma	Partial	[132]
*ARR_2_PB-FGFR1 (JOCK1)*	2003	None	Hyperplasia	No	[134]
*PB-Ras*	2004	None	PIN	No	[136]
*PB-neu*	2006	None	Adenocarcinoma	Yes	[141]
*ARR_2_PB*-*ERG *	2008	None	PIN	Not determined	[142–145]
*ARR_2_PB*-*hepsin*/ *ARR_2_PB-myc *	2010	None	Adenocarcinoma	Partial	[133]

**Table 3:** Traditional knockout models.

Model	Year	Other tissues affected	Highest stage of neoplasia	Castration resistance	References
*RAR*γ**	1993	Seminal vesicles, wide array of developmental defects	Squamous cell metaplasia	Not determined	[147]
*p27*	1996	Hyperplasia of many organs, infertility in females, pituitary tumors, increased overall body size	Hyperplasia & increased stromal cells	Not determined	[149–151]
*Pten*	1998	Gonads, germ cells, lymphoid cells, epidermis, uterus, endometrium, intestine, thyroid, adrenal gland	PIN	Not determined	[153, 154]
*Nkx3.1*	1999	Salivary glands, bulbourethral gland, seminal vesicles	Hyperplasia & dysplasia	Not determined	[155–157]
*Stat5a*	1997	Mammary glands	None	Partial	[158–161]
*Pten × p27*	2001	Endometrium, intestine, thyroid, adrenal gland	Adenocarcinoma	Not determined	[162]
*Pten × Nkx3.1*	2002	Similar to Pten and Nkx3.1 models, though only prostate was reported on	LN metastasis	Yes	[163, 164]
*Pten × p53 *	2009	Similar to Pten model, though only prostate was reported on	HGPIN	Not determined	[165]

**Table 4:** Conditional knockout models.

Model	Year	Other tissues affected	Highest stage of neoplasia	Castration resistance	References
*PSA^Cre^Nkx3.1^flox^*	2002	None	PIN	Not determined	[176]
*PSA^Cre^Pten^flox^*	2005	None	Infrequent LN metastasis	No	[177]
*PB^Cre4^Pten^flox^*	2003	None	LN & lung metastasis	Yes	[178, 179]
*PB^Cre^Rb^flox^*	2004	None	Hyperplasia	Not determined	[180, 181]
*PB^Cre4^LSL*-*Ph15LO*-*1 (FLiMP) *	2006	None	PIN	Not determined	[182]
*PB^Cre4^p53^flox^Rb^flox^*	2006	None	LN, lung, liver & adrenal glands metastasis	Yes	[180, 181]
*PB^Cre4^Apc^flox^*	2007	None	Adenocarcinoma	Yes	[183]
*PB^Cre4^IGF*-*1^flox^*	2008	None	Hyperplasia	No	[184]
*PB^Cre4^Catnb^lox(ex3)^*; *K-ras^LSLV12^*	2009	Bulbourethral gland, preputial gland, and urethral gland metaplasias, testes	Adenocarcinoma	Putative	[185]
*PB^Cre4^Catnb^lox(ex3)^* & *Nkx3.1^Cre^Catnb^lox(ex3)^*	2009	None	HGPIN	Yes	[186]
*PB^Cre4^Brca2^flox^p53^flox^*	2010	None	HGPIN	Yes	[187]
*MMTV^Cre^Catnb^lox(ex3)^*	2003	Skin, salivary glands, epididymis, vas deferens, seminal vesicle, spleen, mammary tissue	Hyperplasia & metaplasia	No	[188]
*MMTV^Cre^Pten^flox^*	2004	Mammary tissue, skin, lymphocytes, oocytes, seminal vesicles, salivary glands	HGPIN	No	[189]
*FSP1^Cre^TGF*β*^flox^*	2004	Fibroblasts, particularly in skin and stomach	PIN	Not determined	[190]
*Nkx3.1^CreERT2^Pten^flox^*	2009	Unknown	Adenocarcinoma	Yes	[16]
